# Anxiety and depression are risk factors for recurrent pregnancy loss: a nested case–control study

**DOI:** 10.1186/s12955-021-01703-1

**Published:** 2021-03-08

**Authors:** Yanxia Wang, Zhaoyan Meng, Jianyin Pei, Liu Qian, Baohong Mao, Yamei Li, Jing Li, Zhirong Dai, Jianing Cao, Chunhua Zhang, Lina Chen, Yuxia Jin, Bin Yi

**Affiliations:** 1 Department of Scientific Research Center of Gansu Provincial Maternity and Child-Care Hospital, No.143. Qilihe North Rd., Lanzhou, 730050 Gansu People’s Republic of China; 2 Department of Perinatal Medicine Center of Gansu Provincial Maternity and Child-Care Hospital, No.143. Qilihe North Rd., Lanzhou, 730050 Gansu People’s Republic of China

**Keywords:** Recurrent pregnancy loss, Anxiety, Depression, Miscarriage, Interaction effect, Risk factors

## Abstract

**Background:**

To evaluate the interaction of depression and anxiety with the development of recurrent pregnancy loss (RPL).

**Methods:**

A nested case–control study involving 2558 participants was conducted with data from the prospective Miscarriage Woman Cohort study between 2017 and 2019 in the province of Gansu, China. The questionnaire data, self-rating anxiety scale and self-rating depression scale were collected after each participant’s first miscarriage. Information on RPL outcomes was obtained from the medical records within the subsequent 2 years. All patients diagosed RPL were recruited as cases whilst a randomly selected group of women with only one miscarriage in the past were recruited as controls. The logistic regression and the interaction effects between anxiety and depression and RPL were analysed.

**Results:**

The prevalence of anxiety (n = 325, 28.7% vs. n = 278, 19.5%) and depression symptoms (n = 550, 48.6% vs. n = 589, 41.3%) for the 1132 RPL cases were higher than 1426 non-RPL controls (*P* < 0.001). After adjusting for possible confounding variables, the odds ratio (OR) value, reflecting the multiplicative interaction, was 1.91 (95% CI 1.50–2.44, *P* < 0.001) for cases with both anxiety and depression symptoms compared with the non-RPL group. The relative excess risk of interaction value, reflecting the additive interaction between anxiety and depression to RPL was 1.15 (95% CI 0.32–4.21). Moreover, the adjusted OR for RPL cases with mild anxiety and severe depression was 2.77 (95% CI 1.07–44.14, *P* < 0.001), for RPL cases with severe anxiety and mild depression was 4.23 (95% CI 1.01–22.21, *P* < 0.001), for RPL cases with severe anxiety and moderate depression was 4.34 (95% CI 1.03–21.28, *P* < 0.001) and for RPL cases with severe anxiety and severe depression was 5.95 (95% CI 1.09–45.09, *P* < 0.05).

**Conclusions:**

Either depression or anxiety alone could increase the risk of subsequent RPL. Anxiety and depression had a synergistic effect after the first miscarriage which increased the development of subsequent RPL disease.

## Introduction

Pregnancy loss or miscarriage is a common disorder in women of child-bearing age. Two or more continuous pregnancy losses before the 24th week of gestation is defined as recurrent pregnancy loss (RPL) [1, 2]. The risk for pregnancy loss or miscrriage was 16%, 25%, 45% and 54% after one to four previous consecutive spontaneous abortions, respectively [3]. RPL causes great harm to couples, especially regarding low level of quality of life in role physical, role emotional, general health, mental health, vitality and social functioning [4]. They had to face the anxiety, ‘subsyndromal depression’, ‘depressive disorder’, and ‘complicated grief’ [5, 6].

Women with a history of pregnancy loss showed more psychological problems during their subsequent pregnancy and lasted 4 months [7]. Moreover, couples have had to face the cumulative effect of RPL with increased exhaustion and pressure exerted by the subsequent pregnancy losses. Saraswat et al. found the threatened miscarriage in the first trimesteris associated with increased incidence of adverse maternal and perinatal outcome [8]*.* The increase of pregnancy-specific stress in the second and third trimester would lead to an increase in the incidence of preterm delivery [9]. It remains to be elucidated whether the reduced RPL rate was the result of lower anxiety and depression after a previous pregnancy loss. If the risk factors were identified, the occurrence of RPL could be prevented. However, there is little evidence to this effect in the relevant literature [10]. There were also few reports on healthy life behavior intervention to prevent RPL by adjusting psychological status and health behaviour [11].

To test the hypothesis: the anxiety and depression may be both the cause and also an adverse effect of RPL. A nested case–control study with data from the prospective Pregnancy Woman Cohort study (PWC) in the province of Gansu was conducted to investigate the relationship between maternal depression and anxiety factors after a miscarriage and the development of RPL.

## Methods

### Study design

This study involving 2558 study participants was conducted with data from the prospective PWC study between June 2017 and June 2019 in the Gansu province of China. The female patients who had miscarriage for the first time, planned to undergo cause examination and continued to have child-bearing desire were enrolled in the prospective PWC study. We intended to explore the weight management of pregnant women and other factors that affect pregnancy outcomes. A face-to-face psychological interview was carried out at that time and the birth outcomes were obtained from the hospital medical record system after 2 years as following. This study was approved by the Ethics Committee of Gansu Provincial Maternity and Child-Care Hospital (REC 2017, GSFY 16).

### Study participants

A pregnancy loss or miscarriage is defined as the spontaneous demise of a pregnancy before 24 weeks of gestation [2]. Women who experienced the losses of two or more pregnancies were defined as RPL cases (their first miscarriage was also counted during the study) [3]. The non-RPL controls were randomly screened out and matched by maternal age. Potential cases and controls were excluded if the woman chose to induce abortion, faced infertility problems, previous ectopic pregnancies, molar pregnancies, stillbirth, neonatal death, suffered from pregnancy complications (i.e. pre-eclampsia, gestational diabetes…), chronic diseases or had a history of psychiatric disorders or addiction and was unavailable for analysis. On matching 1:1 case controls, we re-checked and eliminated those who did not meet the admission criteria and lost follow-up. Thus, a total of 1132 cases (RPL group) and 1426 controls (non-RPL group) were included in this study.

### Data collection

An in-person structured interview was undertaken with the participants after their first miscarriage during the cohort study by a specially trained nurse at the hospital. Information collected during the interview included socioeconomic characteristics (e.g. maternal age, ethnicity, education, occupation, family monthly income) and lifestyle habits before miscarriage (e.g. active or passive smoking status, alcohol consumption, sleep quality and level of physical exercise). Information on the maternal menstrual and reproductive history (e.g. menstrual cycle, previous liveborn; gestational age, time limit of past pregnancy loss, whether or not have embryonic chromosome abnormalities about this miscarriage) were obtained from the participants’ medical records. Follow-up data of the subsequent pregnancy outcomes (e.g. RPL, no pregnancy, or ≥ 24 gestational weeks) were obtained via outpatient department visits and telephone interviews until 30 June 2019. The follow-up rate was 88.2%.

### Measurements

The self-rating anxiety scale (SAS) [12] and the self-rating depression scale (SDS) [13] were used respectively (Chinese versions) to ascertain the women’s true situations regarding depression and anxiety during the first few days after their first miscarriage. The SDS and SAS both contain 20 items, using a point score from the baseline of one. The point scores indicate the following: 1 = ‘none or a little of the time’; 2 = ‘some of the time’; 3 = ‘a good part of the time’; and 4 = ‘most or all of the time’. The original total scores of the SDS and SAS of all women ranged from 20 to 80. The SDS and SAS indices were obtained by multiplying the total score on each questionnaire by 1.25 and converting to a 100-point scale. According to the primary screening diagnostic criteria of Chinese anxiety and depression norms: SAS ≥ 50 and SDS ≥ 53 were defined, respectively, as diagnosed anxiety and depression. The alpha Cronbach for SAS and SDS is 0.82 and 0.78 [14].

### Statistical analysis

Indicators such as age and the family monthly income are artificially classified by the researchers as: ≤ 25, 26–29, 30–34, ≥ 35 and < 2000, 2000–3999, 4000–5999, ≥ 6000 [15]; The quantitative variables of the SAS score and SDS score were converted to qualitative variables by the scale from the book named “Manual of Mental Health rating scale (China)”. Anxiety: mild (score 50–59), moderate (score 60–69) and severe (score ≥ 70); depression: mild (score 53–62), moderate (score 63–72) and severe (score ≥ 72) [14].

A chi-square test was used to evaluate the statistically significant differences between the RPL and control groups. The multiple logistic regression analysis was performed to analyse the relationship between anxiety and depression symptoms from miscarriage and the occurrence of subsequent RPL. The data used for the regression equation were derived from the statistically significant variables and clinically relevant reported data. Maternal age, ethnicity, family monthly income, education, time of miscarriage at baseline, whether they had gave birth to a child, time limit of past pregnancy loss and foetal abnormalities were analysed as the potential confounding factors.

Furthermore, the effects of anxiety interaction with depression of different levels (no/mild/moderate/severe) on RPL was analysed by using "multiple interaction" from the logistic regression model and “addition interaction statistical analysis model” which were reported by Andersson [16]. The odds ratios (OR_A*B_) and 95% confidence intervals (CI_A*B_) was used to analyse the interaction effect. Moreover, the relative excess risk due to interaction (RERI, RERI = OR_AB_ − OR_A_ − OR_B_ + 1) value with 95% CI, the attributable proportion due to interaction (AP) and the synergy index (S) was used to reflect the additive interaction effect, which was more likely to evaluate the biological interaction between risk factors and the disease. Both RERI and AP are positive, and the confidence interval does not contain 0; S > 1, the confidence interval does not contain 1, indicating that they interact on the additive scale and are synergistic. (if RERP and AP are less than 0 and S is less than 1, then it is antagonistic). The results suggest a synergistic effect of anxiety and depression on the occurrence of RPL when the RERI > 0 and the lower limit of 95% CI > 0 [16].

The SPSS software (SPSS Inc., Chicago, IL, USA, version 19.0) and the Excel software from Andersson T. were used to perform the statistical analyses. A *P* value of < 0.05 was considered statistically significant.

## Results

### Sociodemographic characteristics of study participants

The sociodemographic features of the participants are summarised in Table 1. A total of 2558 individuals were enrolled in the study. Among these, 93% of the participants were of the Han nationality, that with the largest proportion of ethnic classification in China. The mean age (standard deviation, SD) of women with and without RPL were 32.6 (3.9) and 32.9 (4.8) years, respectively. There was no statistical difference between two groups by chi-square test (*P* = 0.19). The number of participants with educational qualifications of both high or technical school and college graduates was 57.9% and 62.3% between two groups. With regard to occupation, the numbers of employed women were 79.6% and 80.5%, respectively. The family monthly incomes, alcohol consumption and the time limits of past pregnancy loss showed no significant differences between the two groups (*P* = 0.13). However, there were higher incidences on the embryonic chromosome abnormalities, previous liveborn and active smoking in women with RPL (*P* < 0.05).

**Table 1:** Basic characteristics of the study population in different demographic categories

Characteristics	RPL group (n = 1132)		Control group (n = 1426)		*P* value^*^
	n	(%)	n	(%)	
Age (year)					0.19
≤ 25	38	3.4	59	4.1	
26–29	324	28.6	422	29.6	
30–34	358	31.6	481	33.7	
≥ 35	412	36.4	464	32.5	
Ethnicity (%)					0.20
The Han nationality	1045	92.7	1335	93.6	
Other	87	7.7	91	6.4	
Education					0.09
≤ primary school	59	5.2	64	4.5	
Middle school	418	36.9	473	33.2	
High or technical school	618	54.6	827	58.0	
≥ College graduate	37	3.3	62	4.3	
Occupation					0.57
Employed	901	79.6	1148	80.5	
Housewife	231	20.4	278	19.5	
Monthly income (RMB, Yuan)					0.13
< 2000	333	29.4	360	25.2	
2000–3999	468	41.3	620	43.5	
4000–5999	104	9.2	139	9.7	
≥ 6000	227	20.1	307	21.5	
Time limit of past pregnancy loss					0.76
≤ 12 weeks of gestation	1041	92.0	1316	92.3	
> 12 weeks of gestation	91	8.0	110	7.7	
Embryonic chromosome abnormalities					0.002
No	1109	98.0	1417	99.4	
Yes	23	2.0	9	0.6	
Previous liveborn					0.001
No	877	77.5	1250	87.7	
Yes	255	22.5	176	12.3	
Alcohol consumption					0.87
No	1103	97.4	1388	97.3	
Yes	29	2.6	27	2.7	
Active smoking					0.001
No	1087	96.0	1400	98.2	
Yes	45	4.0	26	1.8	
Passive smoking					0.11
No	979	86.5	1263	88.6	
Yes	153	13.5	163	11.4	

*RPL* recurrent pregnancy loss

*All *P* values derived from Chi-square test

### Depression and anxiety levels before RPL in the two groups

Table 2 shows the association between different depression and anxiety levels of participants and the development of RPL. The total prevalence of self-anxiety symptoms (SAS > 50) were 28.7% and 19.5% in the RPL and control groups, respectively. The total prevalence rates of self-depression symptoms (SD > 53) were 48.6% and 41.3% in the RPL and control groups, respectively. The mild anxiety levels (19.3% vs. 14.1%, *P* < 0.001) and mild depression levels (30.2% vs. 27.7%, *P* < 0.001) after first miscarriage were significantly more common among women with subsequent RPL than in the controls. On further adjustment for potential confounding variables, the adjusted OR (95% CI) comparing the anxiety levels of 60–69 (moderate) and ≥ 70 (severe) with those of no anxiety as the reference were 1.19 (1.01–2.37) and 1.26 (1.04–2.96) in individuals who had subsequent RPL. Similarly, the adjusted OR (95% CI) for RPL comparing depression levels of 53–62 (mild), 63–72 (moderate) and ≥ 72 (severe) with a depression level < 53 as the reference in the non-RPL group were 1.29 (1.13–4.65), 1.64 (1.02–3.35) and 1.40 (1.02–2.92), respectively.

**Table 2:** Anxiety or depression symptoms at baseline in association with occurrence of RPL

Symptoms	Total	RPL group (n = 1132)		Control group (n = 1426)		Crude OR (95% CI)	Adjusted OR^*^ (95% CI)
		n	%	n	%		
Anxiety, SAS	2558						
< 50 (normal)	1955	807	71.3	1148	80.5	1.00	1.000
50–59 (mild)	420	219	19.3	201	14.1	**1.23 (1.10–1.37)**	1.07 (0.39–1.45)
60–69 (moderate)	136	80	7.1	56	3.9	**1.43 (1.16–1.75)**	**1.19 (1.01–2.37)**
≥ 70 (severe)	47	26	2.3	21	1.5	1.31 (0.95–1.81)	**1.26 (1.04–2.96)**
SAS score (Mean ± SD)		42.3 ± 9.7	45.5 ± 10.2				
Depression, SDS	2558						
< 53 (normal)	1419	582	51.4	837	58.7	1.00	1.00
53–62 (mild)	737	342	30.2	395	27.7	**1.10 (1.02–1.19)**	**1.29 (1.13–4.65)**
63–72 (moderate)	365	186	16.4	179	12.6	**1.20 (1.07–1.35)**	**1.64 (1.02–3.35)**
≥ 72 (severe)	37	22	1.9	15	1.1	1.46 (0.98–2.16)	**1.40 (1.02–2.92)**
SDS score (Mean ± SD)		49.6 ± 12.0	52.0 ± 11.5				

Bold values indicate better results than other filtering methods

*RPL* recurrent pregnancy loss, *CI* confident interval, *OR* odds ratio

* adjusted for age, ethnicity, education, family monthly income, active smoking, previous liveborn, and embryonic chromosome abnormalities

### Effects of the anxiety and depression interaction on the development of RPL

The effects of the anxiety–depression interaction on the development of RPL using statistical analysis models are outlined in Table 3. The prevalence rates of comorbid anxiety and depression symptomology were 18.3% and 14.5% in the RPL and control groups, respectively. First, the interaction analysis models were used; the adjusted OR (95% CI) for RPL comparing comorbid anxiety and depression symptomology with no anxiety and depression symptomology as the reference in the non-RPL group were 1.91 (1.50–2.44). Second, the addition interaction analysis models were used; the RERI value was 1.15 (0.32–4.21) and the AP value was 0.25 (0.04–0.82).

**Table 3:** The joint association of anxiety and depression at baseline with occurrence of RPL

Anxiety	Depression	RPL group (n = 1132)		Control group (n = 1426)		Adjusted OR (95% CI)^a^
		n	%	n	%	
No	No	464	41.0	703	49.3	1.00
Yes	No	118	10.4	134	9.4	1.32 (0.98–1.77)
No	Yes	343	30.3	343	24.1	**1.26 (1.04–1.53)**
Yes	Yes	207	18.3	207	14.5	**1.91 (1.50–2.44)**^**b**^
Anxiety + Depression^c^: RERI: 1.15 (0.32, 4.21); AP: 0.25 (0.04, 0.82); S:1.48 (0.52, 4.19);						

Bold values indicate better results than other filtering methods

*RPL* recurrent pregnancy loss, *CI* confidence interval, *OR* odds ratio, *RERI* relative excess risk due to interaction. The RERI between anxiety (A) or depression symptoms (B) to RPL was calculated using the formula: RERI = ORAB − ORA − ORB + 1

^a^Adjusted for age, ethnicity, education, family monthly income, active smoking, previous liveborn, and embryonic chromosome abnormalities;

^b^Adjusted OR (95% CI) of the multiple interaction between anxiety and depression from the logistic regression model

^C^Addition interaction statistical analysis model was used to analyse the effect of anxiety and depression

### The effects of anxiety interaction with depression of different levels on RPL

Table 4 shows the effects of anxiety interaction with depression of different levels (no/mild/moderate/severe) on RPL using interaction analysis methods. After adjustment for all confounding variables, compared with the non-RPL participants without anxiety and depression, the adjusted OR (95% CI) for RPL without anxiety but with mild, moderate or severe depression conditions were 1.16 (0.93–1.46), 1.46 (1.08–1.91) and 1.81 (1.04–3.32), respectively. Furthermore, the adjusted OR (95% CI) for RPL without depression but with mild, moderate or severe anxiety conditions were 1.49 (1.06–2.11), 1.32 (1.09–2.30), 3.25 (1.21–8.12), respectively. Compared with the non-RPL participants without anxiety and depression. The adjusted OR (95% CI) for RPL with mild depression but with mild, moderate or severe anxiety were 1.46 (0.99–2.13), 2.48 (1.22–5.02), 4.23 (1.01–22.21), respectively. The adjusted OR (95% CI) for RPL with severe anxiety and moderate depression conditions were 4.34 (1.03–21.28). The adjusted OR (95% CI) for RPL with severe anxiety and severe depression conditions were 5.95 (1.09–45.09).

**Table 4:** Adjusted odd ratios (95% CI) with occurrence of RPL by joint effects of maternal anxiety and distress

The interaction effect of psychological anxiety and distress	No depression		Mild depression		Moderate depression		Severe depression		*P* for trend	Total OR^a^ (95% CI)
	Case/controls	OR^a^ (95% CI)	Case/controls	OR^a^ (95% CI)	Case/controls	OR^a^ (95% CI)	Case/controls	OR^a^ (95% CI)		
Anxiety										
Normal	464/703	1.00	213/299	1.16 (0.93–1.46)	121/135	**1.46 (1.08–1.91)**	9/11	**1.81 (1.04–3.32)**	–	1.00
Mild	85/86	1.49 (1.06–2.11)	85/80	1.46 (0.99–2.13)	42/33	1.41 (0.82–2.43)	7/2	**2.77 (1.07–44.14)**	< 0.001	**1.57 (1.26–1.97)**
Moderate	27/33	**1.32 (1.09–2.30)**	36/14	**2.48 (1.22–5.02)**	14/9	1.50 (0.59–3.79)	3/0	–	< 0.05	**1.73 (1.19–2.54)**
Severe	6/15	**3.25 (1.21–8.12)**	8/2	**4.23 (1.01–22.21)**	9/2	**4.34 (1.03–21.28)**	3/2	**5.95 (1.09–45.09)**	< 0.05	1.36 (0.72–2.59)
*P* for trend	0.40	< 0.001		< 0.001		< 0.001		< 0.05	< 0.001	< 0.001
Total *OR*^a^ (*95% CI*)		1.00		**1.31 (1.08–1.58)**		**1.57 (1.22–2.00)**		**2.23 (1.11–4.49)**	< 0.05	

Bold values indicate better results than other filtering methods

*RPL* recurrent pregnancy loss, *CI* confidence interval, *OR* odds ratio

^a^Adjusted for age, ethnicity, education, family monthly income, active smoking, previous liveborn, and embryonic chromosome abnormalities

## Discussion

There have been many reports on the pathogeneses of RPL, including chromosomal abnormalities, autoimmune diseases, anti-phospholipid antibodies, endocrinological abnormalities, thrombophilia disorders and uterine abnormalities [1–3]. Nearly half of the aforementioned reasons for pregnancy loss should be explored along with their psychological and psychiatric effects [2, 17]. RPL has a remarkable emotional and psychological influence on women of child-bearing age and their families [2]. To date, studies have focussed primarily on women and their partners’ anxiety and depression after pregnancy loss [5]. However, there is little epidemiological evidence to support that negative psychology is a risk factor for RPL. Are psychological problems risk factors for RPL? Do anxiety and depression have a biological interaction with RPL to induce miscarriage in women? Are the statuses of anxiety and depression mutually causal with the occurrence of RPL? Therefore, we used a nested case–control study including the data of 1132 RPL cases and 1426 non-RPL controls from the prospective PWC study to explore the relationship between anxiety and depression after a miscarriage and the subsequent occurrence of RPL. The symptoms of anxiety and depression after a miscarriage were risk factors for RPL at follow-up. The biological interaction between depression and anxiety symptoms increased the risk of developing RPL.

## Main findings

Women with pregnancy loss or miscarriage commonly develop posttraumatic stress disorder, anxiety, depression and other negative psychological problems [18]. The depressive disorders were accompanied by anxiety during the follow-up. The anxiety and depression levels after the first miscarriage were significantly more common among women with subsequent RPL than in the non-RPL controls (*P* < 0.001). Pregnancy loss or miscarriage interacts with negative psychological situations. The stress effect caused by the first abortion is not transient. Chronic stress can increase maternal anxiety and depression-like behavior.

The study used logistic regression and addition interaction analysis models and found that the adjusted OR (95% CI) for RPL that compared comorbid anxiety and depression symptomology with the absence of anxiety and depression symptomology as the reference in the non-RPL group was 2.788 (1.511–5.144). The RERI value was 1.148 (0.316, 4.212) and AP value was 0.253 (0.038, 0.823). The results suggest a synergistic effect of anxiety or depression on the development of RPL.

### Comparison with other studies

The prevalence of anxiety and depression symptoms being higher in the RPL group than in the control group has been reported by previous literature. Nikcevic et al. [19] reported that almost all women faced grief, anxiety and depression after experiencing a miscarriage, along with the physical recovery. Klier et al. [20] reviewed the related literature and reported that various negative psychological problems would last for almost six months after the loss event. Kagami et al. [21] reported the interaction between the RPL-related negative psychology and unhealthy marital relationships. Mevorach-Zussman et al. [22] found that women with RPL experience showed higher anxiety levels and lower quality of life than healthy women; however, previous literature has not proved that anxiety caused RPL. Kolte et al. [23] pointed out that a high stress level was more prevalent in women with RPL. Depression in women with RPL was more than five times higher than that in healthy women. Koert et al. [24] reported that previous RPL might be a predictor of prenatal and postpartum depression. In conclusion, our conclusion was consistent with that reported in previous literature.

A reciprocal link between anxiety and distress during the pregnancy period has been reported only in a few observational studies. Ramakrishna et al. [25] reported that the prevalence of comorbid anxiety and depression symptomology for women was 13.4% during the postpartum period. Related studies identifying the psychological status correlated with RPL comorbidity are rare. Furthermore, the present study found that the longer the high levels of comorbid anxiety and depression persisted after the pregnancy loss, the occurrence of recurrence of the disease was higher. A interaction was observed between anxiety and depression to different degrees. In other words, the women who had had miscarriages with both depression and anxiety had an excess risk of RPL. This implies that a considerable number of RPL cases may be attributable to the presence of depression and anxiety in the same causal mechanism. One possible explanation for the pregnancy loss or miscarriage events reported in mothers with depressive disorders involves elevated levels of plasminogen activator inhibitor (PAI)-1. PAI-1 was related to placental angiogenesis and vascular remodelling, thus affecting fetal growth [26]. Elevated levels of cortisol also increase the risk of pregnancy complications including RPL. Cortisol related to dopamine levels, which plays a pivotal and role in reward-motivated behavior, and to serotonin levels, which regulates mood, appetite and sleep [27]. In addition, it is reported that the chronic anxiety symptom increased procoagulant activity and decreased fibrinolytic activity, which could increase the risk of adverse obstetric, including RPL [28]. Further research should continue to investigate the underlying mechanistic implications of this interaction.

### Strengths and limitations

This study has many strengths. First of all, our case control study was nested within a prospective miscarriage woman cohort study and the assessment of psychological problems exposure was prior to the diagnosis of RPL, which allowed us to estimate the causal relationship of anxiety and depression exposure on the development of RPL. Second, the analytical method used in the study was the “multiple interaction" from the logistic regression model and “addition interaction statistical analysis model”, the analysis performance is very reliable and comprehensive. Third, the RPL cases were confirmed by medical records rather than self-reported.

The following were the potential limitations of this study. The anxiety and depression symptom data were collected only once after the first miscarriage and before the symptomatic treatment. The levels measured at one point in time may not accurately reflect long-term psychological adjustment. The reliability of the psychological scales was also limited. Due to the limited sample size, the effects of severe anxiety combined with those of severe depression on RPL were not truly reflected. Another challenge was the sensitivity and specificity of the SAS and the SDS compared to using the ‘hospital anxiety and depression scale’ as a screening tool to capture symptoms of anxiety and depression [4]. To comprehensively consider the feasibility of implementation, our study has strengthened the quality control of the process to enhance the credibility of our results.

### Implications for practice and future research

Either depression or anxiety alone could increase the risk of subsequent RPL. Anxiety and depression had a synergistic effect after the first miscarriage which increased the development of subsequent RPL disease.

Care providers should take measures to assist couples who have suffered miscarriage to relieve the distress and anxiety conditions when they encounter a pregnancy loss [24]. Further research should focus on the underlying mechanisms of the interactions between psychological changes and RPL development.

## Conclusion

In summary, the anxiety and depression status after the first miscarriage increased the development of subsequent RPL disease. There was a synergistic effect of anxiety and depression on the incidence of RPL in women who have experienced miscarriage. The findings of our study can enrich the risk factor data on psychological status in recurrent spontaneous abortion in women and may be evidence that the progressive aggravation of anxiety and depressive symptoms may accelerate the deterioration of RPL.

## Data Availability

A minimal set of data is available from the corresponding author on request.

## Abbreviations

RPL: Recurrent pregnancy loss
SAS: Self-rating anxiety scale
SDS: Self-rating depression scale
RERI: The relative excess risk of interaction
